# Toxicity thresholds of nine herbicides to coral symbionts (Symbiodiniaceae)

**DOI:** 10.1038/s41598-021-00921-3

**Published:** 2021-11-04

**Authors:** Magena Marzonie, Florita Flores, Nora Sadoun, Marie C. Thomas, Anais Valada-Mennuni, Sarit Kaserzon, Jochen F. Mueller, Andrew P. Negri

**Affiliations:** 1 Australian Institute of Marine Science, PMB No. 3, Townsville, QLD 4810 Australia; 2 AIMS@JCU: Australian Institute of Marine Science and College of Marine and Environmental Sciences, James Cook University, Townsville, QLD 4811 Australia; 3 Queensland Alliance for Environmental Health Sciences (QAEHS), The University of Queensland, Woolloongabba, QLD 4102 Australia

**Keywords:** Marine biology, Conservation biology, Conservation biology, Environmental impact, Ecology, Ocean sciences

## Abstract

Over 30 herbicides have been detected in catchments and waters of the Great Barrier Reef (GBR) and their toxicity to key tropical species, including the coral endosymbiotic algae Symbiodiniaceae, is not generally considered in current water quality guideline values (WQGVs). Mutualistic symbionts of the family Symbiodiniaceae are essential for the survival of scleractinian corals. We tested the effects of nine GBR-relevant herbicides on photosynthetic efficiency (ΔF/F_m_′) and specific growth rate (SGR) over 14 days of cultured coral endosymbiont *Cladocopium goreaui* (formerly *Symbiodinium* clade C1). All seven Photosystem II (PSII) herbicides tested inhibited ΔF/F_m_′ and SGR, with toxicity thresholds for SGR ranging between 2.75 and 320 µg L^−1^ (no effect concentration) and 2.54–257 µg L^−1^ (EC_10_). There was a strong correlation between EC_50_s for ΔF/F_m_′ and SGR for all PSII herbicides indicating that inhibition of ΔF/F_m_′ can be considered a biologically relevant toxicity endpoint for PSII herbicides to this species. The non-PSII herbicides haloxyfop and imazapic did not affect ΔF/F_m_′ or SGR at the highest concentrations tested. The inclusion of this toxicity data for Symbiodiniaceae will contribute to improving WQGVs to adequately inform risk assessments and the management of herbicides in tropical marine ecosystems.

## Introduction

### Pesticides in tropical marine ecosystems

Pesticide contamination from coastal agriculture affects tropical and sub-tropical nearshore marine environments globally^1–5^. In Australia, large-scale monitoring and reporting programs for the Great Barrier Reef (GBR) aim to inform the management of pesticide loads that enter the GBR and its catchments^6^. There have been at least 55 pesticides detected in the GBR catchment area (GBRCA)^7^ and 99.8% of water samples retrieved from 2011 to 2015 contained detectable concentrations of pesticides and pesticide mixtures^8^. Herbicides are the most frequently detected pesticides (> 30)^7^ and reach peak loads and concentrations during the summer wet season^9,10^. Due to their persistence, some herbicides are detected year-round, resulting in chronic exposure to freshwater and marine organisms^11^. The five most frequently detected herbicides in the GBRCA (termed priority herbicides) are the Photosystem II (PSII) inhibitors diuron, ametryn, atrazine, tebuthiuron, and hexazinone^12,13^. This herbicide class acts by binding to the D1 protein in PSII, causing oxidative damage, reducing photosynthetic capacity and leading to the chronic impairment of cellular function^14^. Regulations have been implemented to reduce the environmental concentrations of these priority herbicides, leading to increased application of ‘alternative’ PSII and non-PSII herbicides in coastal agriculture^15,16^. While alternative herbicides are detected frequently in the environment, there are still limited data available on their transport, persistence and toxicity to non-target species^17^.

### Herbicide impacts on tropical marine organisms

PSII herbicides can negatively impact a wide range of tropical marine phototrophs including seagrasses^18,19^, crustose coralline algae^20^, *Halimeda*^21^, foraminifera^22^ and marine microalgae^23,24^. Scleractinian corals are foundational invertebrates that provide key habitat and structure for tropical reefs and residing organisms. Corals are susceptible to herbicides as they rely on endosymbiotic dinoflagellates, Symbiodiniaceae (that reside in the coral host tissue), to provide up to 90% of their nutritional requirements through photosynthetic pathways^25^. The blockage of electron transport in endosymbiotic algae by PSII herbicide exposure results in some of the energy that would normally drive electron transport for primary production instead being emitted as fluorescence^26,27^. Consequently, pulse amplitude modulation (PAM) fluorometry has been used in several studies as a highly sensitive and non-destructive technique to quantify sub-lethal effects of PSII herbicides on photosynthetic efficiency (as effective quantum yield: ΔF/F_m_′) in light adapted corals^28^. The inhibition of ΔF/F_m_′ by PSII herbicides is reversible^27,28^, but high concentrations or chronic exposures can lead to the breakdown of symbiosis in corals (bleaching)^27,29,30^ and decreased reproductive output and mortality^29^.

While there has been much focus on the effects of herbicides on Symbiodiniaceae within their coral hosts, these dinoflagellates also have a free-living phase (0.1% of global phytoplankton reads in the open ocean^31^), which act as a critical reservoir for uptake into newly settled aposymbiotic coral recruits and corals recovering from thermal bleaching events^32,33^. Therefore, effects of herbicide contamination on the health or abundance of free-living Symbiodiniaceae could have important flow-on effects to the establishment of symbiotic associations which are critical for coral health. Several studies have demonstrated inhibition of ΔF/F_m_′ by PSII herbicides in freshly isolated or cultured Symbiodiniaceae and that sensitivity differs between species and increases with temperature^34–36^. Impairment of ΔF/F_m_′ by PSII herbicides is highly correlated with reduced growth in several tropical marine microalgae^23,37,38^; however, it is unclear whether inhibition of ΔF/F_m_′ translates to similar whole organism effects in Symbiodiniaceae.

### Improving the applicability of water quality guideline values for herbicides in tropical marine ecosystems

Reliable water quality guideline values (WQGVs) are required to assess the potential risks posed by herbicides and mixtures of herbicides, frequently detected in tropical marine waters including the GBR^8,12^. Conventionally, concentrations of individual herbicides detected in the field are compared against the relevant WQGV for that herbicide. For example, time averaged concentrations of up to 0.778 µg L^−1^ diuron, obtained over a month-long passive sampler deployment, have been detected in the latest GBR monitoring program^12^, a value that exceeds the proposed PC99 WQGV for diuron (0.43 µg L^−1^; protection concentration for 99% of species)^39^. Importantly, measured concentrations of herbicide mixtures can be assessed against a risk metric which predicts the proportion of species potentially affected by that mixture. The metric applied in GBR herbicide monitoring is the multi-substance Potentially Affected Fraction (ms-PAF)^40^, and more exceedances of WQGVs are reported using this approach which accounts for all herbicides present in the environment^12^. The ms-PAF method requires high quality WQGVs for all herbicides detected in the environment; however, several alternative herbicides do not have reliable WQGVs and their contribution to risk can be difficult to assess. The Australian and New Zealand guidelines currently include WQGVs for nine herbicides: the five priority PSII herbicides and four alternative herbicides (2,4-D, bromacil, MCPA, and simazine), but many of these are of low reliability due to lack of toxicity data^41^. Their relevance to tropical marine habitats is also uncertain as the majority of these WQGVs have been derived from toxicity data from freshwater temperate species^23,41^. Marine proposed guideline values (PGVs) have recently been proposed for 20 herbicides: 2,4-D, ametryn, bromacil, diuron, fluometuron, fluroxypyr, haloxyfop, hexazinone, imazapic, isoxaflutole, metribuzin, MCPA, simazine, tebuthiuron, pendimethalin, prometryn, propazine, terbutryn, triclopyr, terbuthylazine^39,42,43^ but the majority are of low to moderate reliability due to lack of suitable marine toxicity data.

More toxicity data are required to improve WQGVs for alternative herbicides detected in tropical marine environments, especially for species of critical ecological value such as corals and their symbionts. Here, we developed a 14-day chronic growth inhibition test for cultures of the free-living coral symbiont *Cladocopium goreaui* to determine the toxicity thresholds for nine herbicides detected in the GBR and the GBRCA. *C. goreaui* was chosen as a suitable test species as it is common in tropical benthic environments and has a relatively rapid growth rate in comparison to many other Symbiodiniaceae^35,44^. The PSII herbicides diuron, bromacil, hexazinone, metribuzin, propazine, simazine and tebuthiuron, as well as the non-PSII herbicides haloxyfop and imazapic were chosen in consultation with the Water Quality and Investigation Team at the Queensland Department of Environment and Science and indicate current toxicity data gaps for the improvement of WQGVs. The effects on specific growth rate (SGR) and ΔF/F_m_′ were investigated^23,37^. The chronic SGR toxicity thresholds (10% effect concentrations (EC_10_) and no effect concentrations (NEC)) represent ecologically relevant endpoints required for inclusion in deriving WQGVs^45^. Correlations between ΔF/F_m_′ and SGR effect concentrations (EC_50_) would further validate the application of PAM fluorometry as a sensitive tool to measure sub-lethal stress in this ecologically important coral symbiont.

## Results

### Physico-chemical test conditions

Physico-chemical parameters were consistent for each test: dissolved oxygen (7.8 ± 0.3 mg L^−1^), pH (7.8 ± 0.5) and salinity (32.5 ± 0.7 psu) (mean ± SD, n = 152), temperature (27 ± 0.6 °C) and light intensity (71 ± 8 µmol photons m^−2^ s^−1^) (mean ± SD, n = 7). All data can be found in Table S2.

### Bioassay performance

*Cladocopium goreaui* exhibited consistent exponential growth in control treatments ranging from 0.0877 to 0.163 SGR day^−1^ among experiments (which were all conducted on separate occasions) (Table 1). There was no effect of carrier solvent (DMSO and ethanol) on SGR (t-test: F_IMK-DMSO_ (1, 3) = 1.185; p = 0.356 and F_IMK-EtOH_ (1, 4) = 0.529; p = 0.507). The effective quantum yield ΔF/F_m_′ was also consistent across control treatments (ΔF/F_m_′ = 0.35 ± 0.04). The reference toxicant diuron (6 μg L^−1^) applied in each toxicity test inhibited SGR by 64.9% ± 3.6% (mean ± SD, n = 44) and ΔF/F_m_′ by 92.8% ± 1.1% (mean ± SD, n = 22).

**Table 1:** Assay performance of solvent control and reference toxicants (6 μg L^−1^ diuron) for each of the nine herbicides tested over 14 days (SGR, n = 4–6 reps; ΔF/F_m_′, n = 3).

Herbicide	Specific growth rate (SGR day^−1^)		Effective quantum yield (ΔF/F_m_′)	
	Control	Ref. Inh. (%)	Control	Ref. Inh. (%)
Diuron	0.148 ± 0.0046	72 ± 3.4	0.329 ± 0.049	96 ± 2.4
Bromacil	0.123 ± 0.011	44 ± 3.1	0.386 ± 0.016	94 ± 4.3
Hexazinone	0.123 ± 0.0066	85 ± 5.8	0.354 ± 0.027	NA
Metribuzin	0.127 ± 0.013	77 ± 5.0	0.356 ± 0.032	NA
Propazine	0.115 ± 0.0050	36 ± 0.7	0.395 ± 0.010	91 ± 2.1
Simazine	0.113 ± 0.011	100 ± 12	0.342 ± 0.053	100 ± 0.00
Tebuthiuron	0.152 ± 0.0064	46 ± 4.6	0.358 ± 0.023	95 ± 5.0
Haloxyfop	0.112 ± 0.0043	49 ± 3.2	0.357 ± 0.043	82 ± 0.1
Imazapic	0.0952 ± 0.0098	38 ± 4.9	0.371 ± 0.020	95 ± 5.3

Mean ± SD. Ref. Inh. (%) denotes percent inhibition of reference toxicant relative to control.

NA  denotes data not measured.

### Toxicity of PSII herbicides: specific growth rate

**Figure 1:**
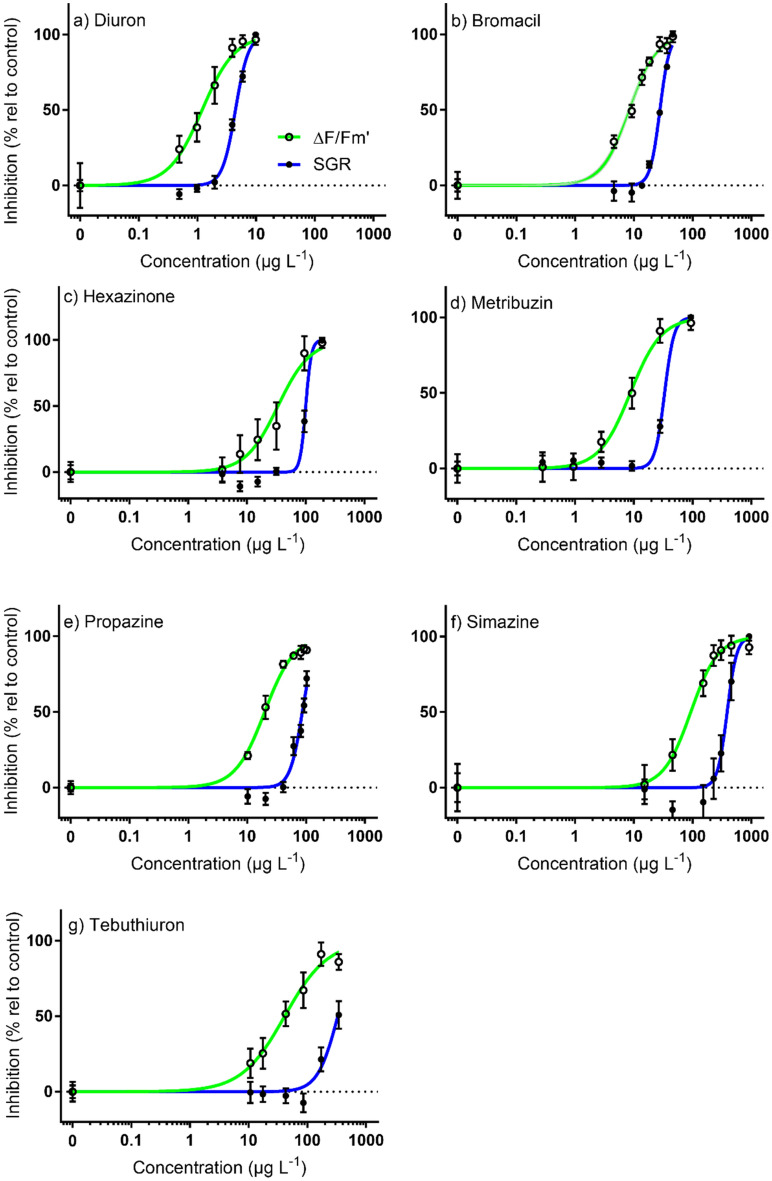
Concentration–response curves for *Cladocopium goreaui* on the relative percent inhibition of 14-day specific growth rate (SGR; closed circle, mean ± SD) and effective quantum yield (ΔF/F_m_′; open circle, mean ± SD) following herbicide exposure to (**a**) diuron; (**b**) bromacil; (**c**) hexazinone; (**d**) metribuzin; (**e**) propazine; (**f**) simazine; and (**g**) tebuthiuron at increasing concentrations. Sigmoidal, 4-parameter curve fit (solid line). All concentrations in µg L^−1^ (n = 4–6 per test; error bars not visible are smaller than symbol).

**Figure 2:**
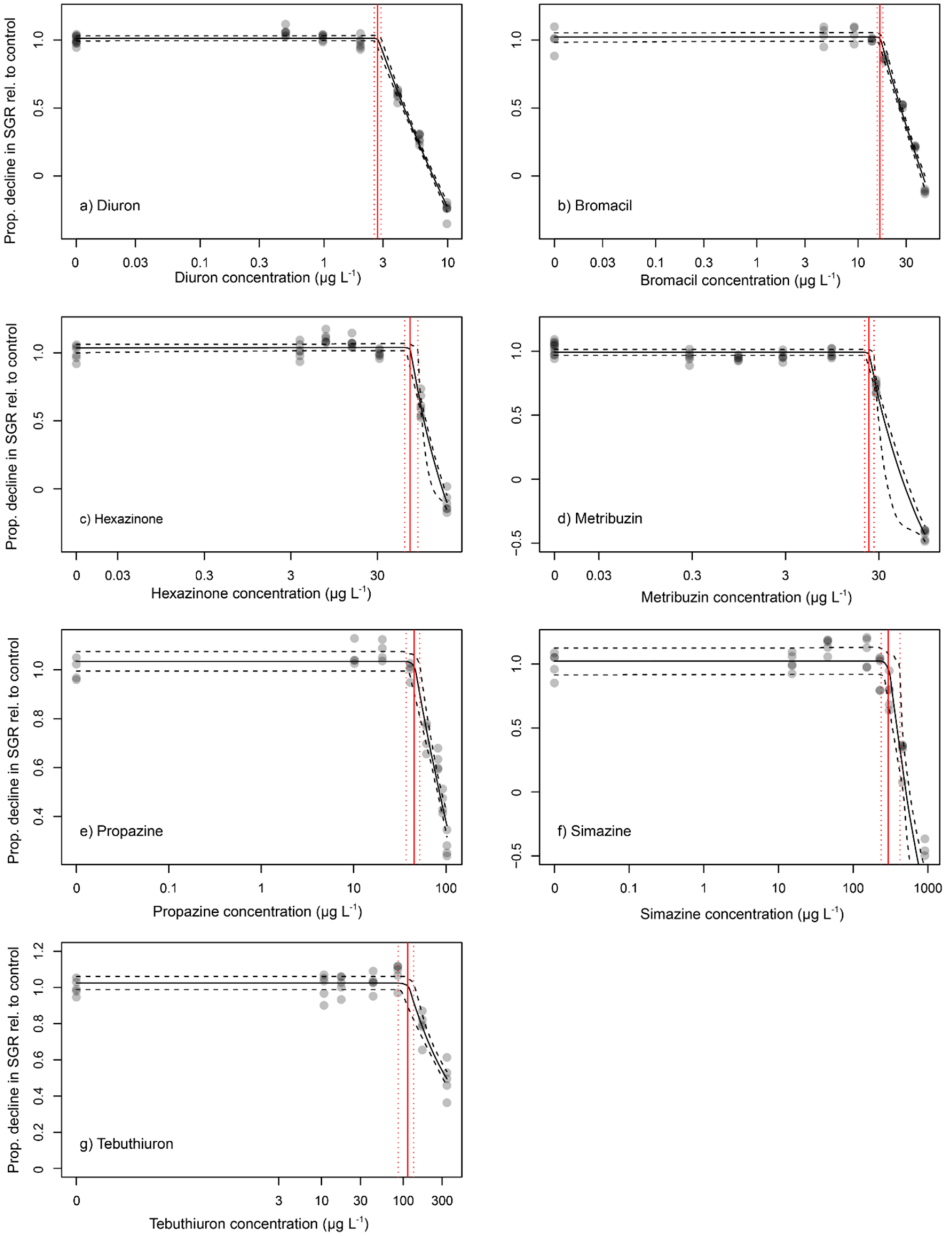
Proportional decline of specific growth rate (SGR) for *Cladocopium goreaui* relative to control treatment (solid black line) and 95% confidence intervals (black dashed line) to derive no effect concentration (NEC) (red line) and 95% confidence interval (red dashed line) of (**a**) diuron; (**b**) bromacil; (**c**) hexazinone; (**d**) metribuzin; (**e**) propazine; (**f**) simazine; and (**g**) tebuthiuron from Bayesian non-linear gaussian model fits. All concentrations in µg L^−1^ (n = 4–6 per test).

The inhibition of SGR by PSII herbicides increased with concentration (Fig. 1). Concentrations of each herbicide that inhibited 10% and 50% of SGR (EC_10_ and EC_50_, respectively), as well as no effect concentrations (NEC) in *C. goreaui* are listed in Table 2. All PSII herbicides were toxic but exhibited a broad range of potencies. *C. goreaui* was most sensitive to the reference herbicide diuron (EC_50_ = 4.45 μg L^−1^) and least sensitive to simazine (EC_50_ = 387 μg L^−1^) (Table 2). All relative potencies were proportionally lower than diuron ranging from bromacil (ReP = 0.16) to simazine (ReP = 0.012). Slopes for fitted concentration–response curves were similar for the PSII herbicides ranging between 2.5 and 5.4 (R^2^ ≥ 0.83), while hexazinone had a much greater slope (Slope = 9.1, R^2^ = 0.97) (Fig. 1). The order of toxicity based on EC_50_ values were: diuron > bromacil > metribuzin > propazine > hexazinone > tebuthiuron > simazine (Table 2). The EC_10_ and NEC toxicity thresholds for SGR followed a similar order of toxicity (Table 2, Figs. 1, 2).

**Table 2:** No effect concentration values (NEC, Fig. 2) and effect concentration values (EC_10_ and EC_50_, Fig. 1) for growth inhibition (SGR) and photoinhibition (ΔF/F_m_′) of *Cladocopium goreaui* over 14 days to each herbicide.

Herbicide	Endpoint	SGR	ΔF/F_m_′	SGR: ΔF/F_m_′ ratio
Diuron	NEC	2.75 (2.56–2.93)		
	EC_10_	2.54 (2.34–2.75)	0.29 (0.26–0.33)	8.76
	EC_50_	4.45 (4.31–4.59)	1.20 (1.15–1.26)	3.71
	*ReP*	*1*	*1*	
Bromacil	NEC	16.6 (15.4–20.6)		
	EC_10_	18.3 (16.9–19.9)	2.54 (2.29–2.82)	7.20
	EC_50_	27.7 (26.7–28.7)	8.36 (8.01–8.69)	3.31
	*ReP*	*0.16*	*0.14*	
Hexazinone	NEC	71.7 (63.4–91.0)		
	EC_10_	78.7 (57.8–92.0)	8.36 (7.14–9.80)	9.41
	EC_50_	100 (96.1–141)	33.8 (30.6–37.6)	2.96
	*ReP*	*0.045*	*0.036*	
Metribuzin	NEC	23.6 (21.3–27.5)		
	EC_10_	22.3 (16.2–25.9)	2.31 (2.08–2.56)	9.65
	EC_50_	33.5 (30.2–50.4)	8.75 (8.39–9.12)	3.83
	*ReP*	*0.13*	*0.14*	
Propazine	NEC	45.1 (37.0–51.1)		
	EC_10_	50.8 (44.8–57.4)	5.42 (4.94–5.95)	9.37
	EC_50_	86.5 (83.0–90.1)	18.7 (18.0–19.5)	4.63
	*ReP*	*0.052*	*0.064*	
Simazine	NEC	320 (234–452)		
	EC_10_	257 (226–294)	28.8 (23.9–35.3)	8.92
	EC_50_	387 (361–416)	93.3 (84.6–102)	4.15
	*ReP*	*0.012*	*0.013*	
Tebuthiuron	NEC	107 (84.6–136)		
	EC_10_	138 (108–173)	6.37 (4.79–8.50)	21.7
	EC_50_	331 (300–NA)	41.0 (36.3–46.3)	8.07
	*ReP*	*0.013*	*0.029*	
Haloxyfop	NEC	> 2980		
	EC_10_	> 2980	> 2980	NA
	EC_50_	> 2980	> 2980	NA
	*ReP*	*NA*	*NA*	
Imazapic	NEC	> 165,000		
	EC_10_	> 165,000	> 165,000	NA
	EC_50_	> 165,000	> 165,000	NA
	*ReP*	*NA*	*NA*	

Concentrations are reported in µg L^−1^ (95% confidence intervals). Relative equivalent potencies (*ReP)* of each herbicide are derived by comparing SGR EC_50_ of each herbicide to the reference herbicide diuron.

Calculated ReP values in italics to differentiate from modelled no effect and effect concentration values.

NA  denotes data not measured.

### Toxicity of PSII herbicides: effective quantum yield

The order of toxicity of PSII herbicides on ΔF/F_m_′ in *C. goreaui* was the same as per SGR (Table 2). However, ΔF/F_m_′ was a more sensitive endpoint: diuron EC_50_ = 1.20 μg L^−1^ and simazine EC_50_ = 93.3 μg L^−1^ (Table 2). Fitted concentration–response curves (Fig. 1) had similar shapes, with slopes for all PSII herbicides ranging from 1.2 to 1.9 (R^2^ ≥ 0.86). Relative potencies of each herbicide relative to diuron ranged from 0.013 to 0.14 (Table 2). The EC_10_ and NEC thresholds for ΔF/F_m_′ inhibition followed a similar order of toxicity to inhibition of SGR (Table 2, Figs. 1, 2).

### Toxicity of non-PSII herbicides

**Figure 3:**
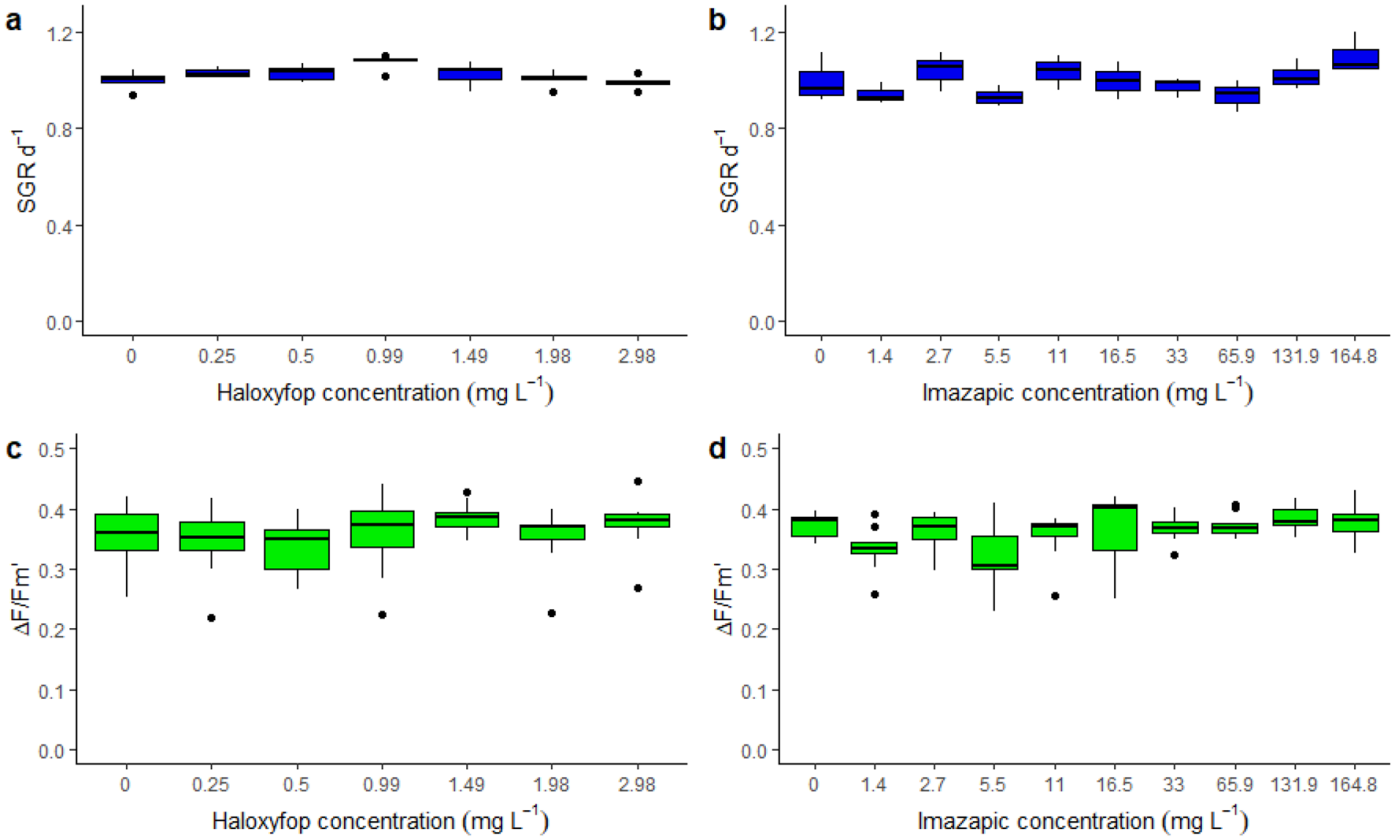
Response of *Cladocopium goreaui* to alternative non-PSII herbicides, haloxyfop and imazapic. Boxplots of the specific growth rate (SGR day^−1^) and effective quantum yields (ΔF/F_m_′) in response to haloxyfop (**a**,**c**) and imazapic (**b**,**d**). Note: all concentrations here are in mg L^−1^.

Both non-PSII herbicides, imazapic and haloxyfop, failed to inhibit growth or ΔF/F_m_′ at the highest concentrations of 165,000 μg L^−1^ and 2980 μg L^−1^, respectively (Table 2; Fig. 3). Higher concentrations of imazapic and haloxyfop were unable to be tested as they altered the pH of the IMK media below an acceptable range.

### Relationship between growth and effective quantum yield inhibition

**Figure 4:**
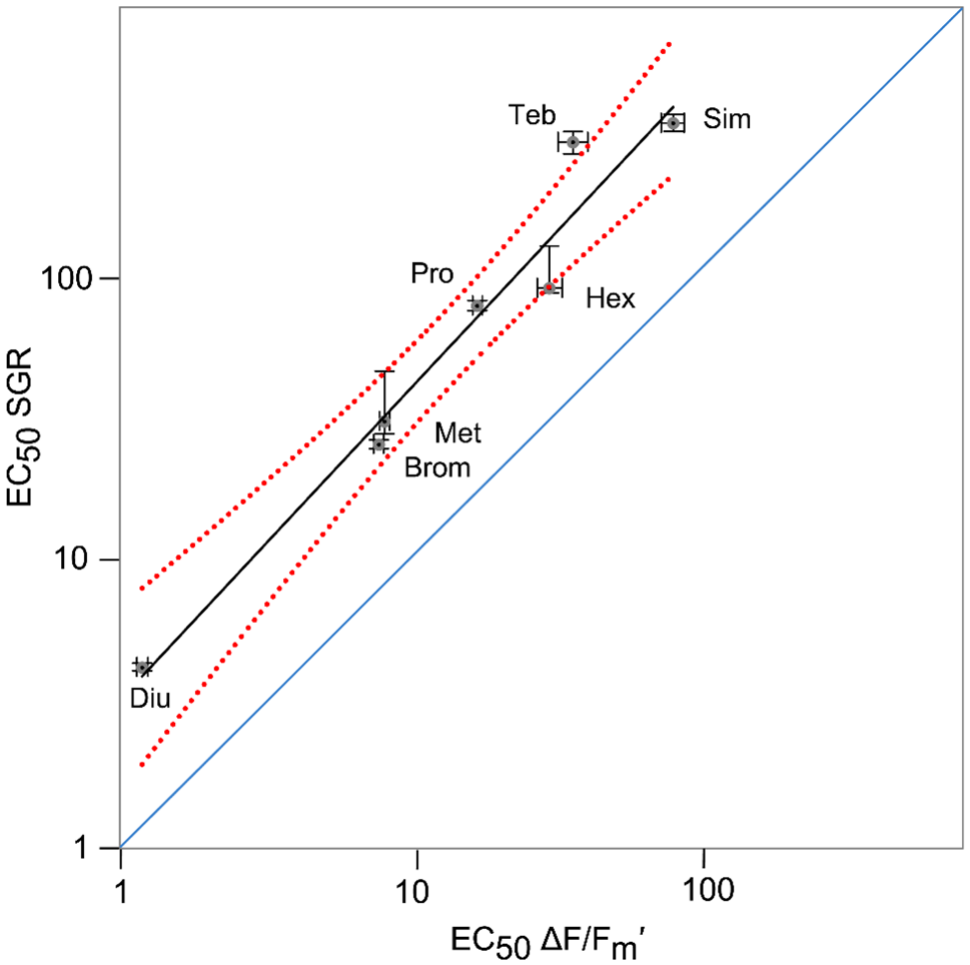
Linear relationship (black line) between inhibition of effective quantum yield (ΔF/F_m_′) and inhibition of specific growth rate (SGR) in *Cladocopium goreaui* by PSII herbicides (dashed red lines 95% confidence bands). Error bars are 95% CI for each EC_50_ value. Blue line indicates 1:1 relationship. *Diu* - diuron, *Brom* - bromacil, *Hex -* hexazinone, *Met* - metribuzin, *Pro* - propazine, *Sim* - simazine, *Teb* - tebuthiuron.

There was a linear relationship between growth inhibition and ΔF/F_m_′ inhibition of PSII herbicides on *C. goreaui* (slope = 4.45; R^2^ = 0.83; Fig. 4). SGR: ΔF/F_m_′ ratios ranged between 2.96 and 4.63, with the exception of tebuthiuron (SGR : ΔF/F_m_′ = 8.10) (Table 2).

## Discussion

### Effects of PSII herbicides on growth

Each of the PSII herbicides inhibited SGR and ΔF/F_m_′ in the cultures of the coral symbiont *C. goreaui* over 14 days. This was expected as the PSII herbicides all bind to the D1 protein site in PSII which is common across phototrophs^46^. Despite the shared mode of action there was a > 80-fold difference in potency between the most toxic herbicide diuron and the least toxic herbicide simazine (comparison of SGR EC_50_s). There was no relationship between toxicity and chemical class. For example, diuron and tebuthiuron are both phenylureas but the toxicity of diuron was > 70-fold greater than tebuthiuron. Substantial differences in toxicity were also evident among the triazines (simazine and propazine) and triazinones (metribuzin and hexazinone). There was also no relationship between EC_50_ values (Table 2) and water solubility or log K_OW_ (Table S1), indicating that transport across cell walls and membranes and/or accumulation within the cells did not have a major influence on relative potency. Instead, the toxicity of each herbicide is most likely related to the specific affinity (steric compatibility and binding properties) between each herbicide and the Q_B_ binding site on the D1 protein in PSII^47^.

Direct comparisons of toxicity between species should be made with caution due to differences in experimental conditions; however, comparing the EC_50_s against datasets with several species’ studies carried out under similar conditions can provide insights into the relative sensitivity of *C. goreaui* compared with other species. Diuron, applied in this study as a reference toxicant, is the most studied PSII herbicide with respect to effects on marine microalgal growth. The growth inhibition EC_50_ values for 17 species range between 3.4 and 110 μg L^−1^ diuron^23,38,39,43,48^, indicating cultured *C. goreaui* is among the most sensitive. The toxicity of herbicides to two other microalgal species *Rhodomonas salina*^23^ and *Chaetoceros muelleri*^38^ were tested in the same laboratory under similar conditions (Table 3). Comparisons between EC_50_ values reveal broadly similar sensitivities to each PSII herbicide (within threefold differences) except for hexazinone, which was > tenfold more toxic to the cryptophyte *R. salina* than *C. goreaui.*

**Table 3:** Comparison among toxicity thresholds of *Cladocopium goreaui, Rhodomonas salina* and *Chaetoceros muelleri*.

Species	*Cladocopium goreaui*	*Rhodomonas salina*	*Chaetoceros muelleri*
Phylum	Dinoflagellata	Cryptophyta	Bacillariophyta
Diuron	4.45 (4.31–4.59)	6.27 (6.02–6.54)	12.4 (11.8–13.0)
Bromacil	27.7 (26.7–28.7)	19.3 (17.7–21.0)	
Hexazinone	100 (96.1–141)	8.50 (7.99–9.06)	
Metribuzin	33.5 (30.2–50.4)	13.4 (12.3–14.5)	
Propazine	86.5 (83.0–90.1)	188 (177–201)	98.2 (91.7–105)
Simazine	387 (361–416)	184 (173–195)	
Tebuthiuron	331 (300–NA)	112 (106–119)	187 (179–195)
Haloxyfop	> 2980	> 3700	> 4570
Imazapic	> 165,000	790,000 (760,000–825,000)	

Effect concentration values (SGR EC_50_) with 95% confidence intervals derived for *Cladocopium goreaui, Rhodomonas salina* and *Chaetoceros muelleri* exposed to herbicides. All experiments were conducted under similar conditions in the same laboratory. Concentrations are reported in µg L^−1^. EC_50_ values for *Rhodomonas salina* obtained from Thomas et al.^23^ and *Chaetoceros muelleri* from Thomas et al.^38^.

### Toxicity of non-PSII herbicides on growth

The two non-PSII herbicides haloxyfop and imazapic had no observable effect on SGR in *C. goreaui.* This insensitivity to the non-PSII herbicides is consistent with other marine microalgae *R. salina*^23^, *C. muelleri*^38^ (Table 3) and *Navicula* spp.^49^. The phenoxy herbicide haloxyfop inhibits the enzyme acetyl-CoA carboxylase (ACCase inhibitor) and blocks production of lipids and fatty acids^42,50^. ACCase inhibitors such as haloxyfop act on homomeric (eukaryotic) ACCases and sequences for homomeric ACCases have been reported in dinoflagellates^51^, indicating the potential for activity. However, the herbicidal activity of haloxyfop-p-methyl also depends on its hydrolysis within a plant and this may not occur within *C. goreaui*^42^*.* Furthermore, Thomas et al.^23^ proposed that the active hydrolyzed form of haloxyfop which contains a carboxyl moiety may bind calcium (Ca^2+^) and/or magnesium (Mg^2+^) ions in seawater^52^ which could affect activity directly or help to stabilize the herbicide at the surface water:air interface^53^. Like haloxyfop, imazapic also has a carboxyl group which may explain its low toxicity to *C. goreaui* cultured in seawater. Furthermore, acetohydroxy acid synthase (AHAS or ALS), the target enzyme for imazapic, has not yet been reported in dinoflagellates^54^.

### Effects of herbicides on photosynthetic efficiency

All PSII herbicides inhibited effective quantum yield (ΔF/F_m_′), which is proportional to photosynthetic efficiency at a given light intensity^55^, in cultured Symbiodiniaceae. Inhibition of ΔF/F_m_′ by the seven PSII herbicides was on average 4.4 times more sensitive than SGR based on EC_50_ ratios (Table 2) and the linear regression slope (Fig. 4). The ratio of inhibition of SGR: ΔF/F_m_′ is comparable to other tropical marine microalgae: *R. salina* (average SGR: ΔF/F_m_′ = 4.3 for 7 PSII herbicides)^23^; *Chaetoceros muelleri* (3.0 for 3 PSII herbicides)^38^; *Navicula* spp. (1.5 for 3 herbicides) and *Nephroselmis pyriformis* (1.3 for 3 herbicides)^49^. Although growth in microalgae is directly dependent on photosynthesis the relationship with inhibition of ΔF/F_m_′ by PSII herbicides may not necessarily be 1:1 since microalgae may draw upon their current resources to continue growth, and some (including Symbiodiniaceae) can adjust to reduced photosynthesis by increasing reliance on heterotrophy^56^. Differences in ΔF/F_m_′ : SGR between studies and species may also be due to light intensity which can affect both ΔF/F_m_′ and SGR^57^, and the nutritional status of cells which is dependent on the composition of the growth media and may change depending on the duration of exposure^58^. Interestingly, when based on EC_10_s the average ratio of SGR: ΔF/F_m_′ was 10.7, indicating an even greater sensitivity of ΔF/F_m_′ to herbicides relative to SGR at lower herbicide concentrations (also reflected by the differences in slopes of the concentration response curves for both parameters (Fig. 1)). The reason for this is unclear; however, relative sensitivities between these endpoints may change with exposure duration. For example, inhibition of ΔF/F_m_′ by PSII herbicides is relatively consistent after it reaches a rapid maxima^27^, while effects of PSII herbicides on SGR may continue to decline over the course of the exposure period (and the nature of this relationship is likely to change with light intensity^57^). The insensitivity of ΔF/F_m_′ in *C. goreaui* to the non-PSII herbicides haloxyfop and imazapic was expected as their modes of action do not block electron transport in PSII, hence there would be no increase in chlorophyll *a* fluorescence^57^. This result was consistent with other tropical marine microalgae *R. salina*^23^, *C. muelleri*^38^ (Table 3) and *Navicula* spp.^49^.

The effects of PSII herbicides on ΔF/F_m_′ have been assessed for both cultured and symbiotic (*in hospite*) forms of Symbiodiniaceae. EC_50_ values have been reported for Symbiodiniaceae *in hospite* with corals for the PSII herbicides: ametryn, diuron, hexazinone, atrazine, simazine, tebuthiuron and irgarol 1051^20,27,28,59^, diuron and hexazinone in a jellyfish^60^ and diuron in an anemone^61^. However, the species of Symbiodiniaceae *in hospite* was only identified in one instance^20^. A comparison of the EC_50_s for diuron among cultured and *in hospite* Symbiodiniaceae (Table 4) shows relatively consistent values among and between these groups. Given that experimental conditions, especially light intensity^22,57^, can affect inhibition of ΔF/F_m_′ in microalgae, direct comparisons are difficult between studies. However, one study found *Durusdinium trenchii* to be twice as sensitive to *C. goreaui* under identical culture and measurement conditions^35^. It was hypothesized that differences in sensitivity to diuron among Symbiodiniaceae types may be due to the reported differences in capacity to repair damaged PSII reaction centres affected by herbicide-mediated photoinhibition^62^. The diuron EC_50_ for *C. goreaui* in the current study was the lowest reported for this species and, while this may be due to differences in experimental conditions, the comparatively long herbicide exposures (14 days) may also play a role, allowing for a build-up of damage to PSII (chronic photoinhibition) and leading to a depression of ΔF/F_m_′^63^. Nevertheless, the strong and consistent relationships between ΔF/F_m_′ and SGR and between Symbiodiniaceae species for multiple PSII herbicides supports the measurement of ΔF/F_m_′ inhibition in marine microalgae as a valuable and biologically relevant toxicity endpoint for PSII herbicides (but should not be applied to herbicides with other modes of action).

**Table 4:** Comparison of EC_50_ values derived for ΔF/F_m_′ in Symbiodiniaceae exposed to diuron*.* EC_50_ values for ΔF/F_m_′ in Symbiodiniaceae in cultured and freshly isolated cells and in symbiosis (*in hospite*). EC_50_ in µg L^−1^.

Symbiodiniaceae species	Culture or symbiotic host (exposure duration)	EC_50_	References
**Isolated cells**			
*Cladocopium goreaui (formerly Symbiodinium Clade C1)*	Culture (14 days)	1.2	This study
	Culture (2 days)	7.4	^34^
	Culture (1 day)	1.4	^11^
	Culture (1 day)	2.1	^35^
*Durusdinium trenchii (formerly Symbiodinium Clade D)*	Culture (1 day)	1.1	^35^
Unknown, isolated from coral *Stylophora pistillata*	Freshly isolated (10 min)	5.5	^27^
**In hospite**			
*Cladocopium* C2 *(formerly Symbiodinium Clade C2)*	Coral: *Acropora millepora* (4 days)	2.9	^20^
Unknown	Coral: *Seriatopora hystrix* (10 h)	2.3	^28^
Unknown	Coral: *Acropora formosa* (10 h)	2.7	^28^
Unknown	Coral: *Acropora formosa* (10 h)	5.1	^27^
Unknown	Coral: *Montipora digitata* (10 h)	5.9	^27^
Unknown	Coral: *Porites cylindrica* (10 h)	4.3	^27^
Unknown	Anemone: *Exaiptasia pallida* (2 days)	8	^61^
Unknown	Jellyfish: *Cassiopea maremetens* (7 days)	1.4	^64^

### Relevance of herbicide sensitivity of free-living Symbiodiniaceae

The apparent lack of differences in sensitivity to diuron between cultured, freshly isolated and *in hospite* Symbiodiniaceae, along with the rapid onset of ΔF/F_m_′ inhibition reported for symbiotic corals^27^, indicates the multiple membrane layers of the coral host provide little barrier to diuron reaching intracellular Symbiodiniaceae. There were also similarities in the sensitivity of cultured *C. goreaui* to EC_50_s reported for coral-hosted Symbiodiniaceae: hexazinone 8.8 µg L^−1^^28^ and 14 µg L^−1^^20^; simazine 150 µg L^−1^ and tebuthiuron 175 µg L^−1^^27^. These comparisons demonstrate a consistency of access and binding of PSII herbicides to the D1 protein within Symbiodiniaceae, regardless of whether the dinoflagellate is in its symbiotic or free-living form and supports the application of cultured *C. goreaui* for assessing the toxicity of PSII herbicides.

The exposure of PSII herbicides to Symbiodiniaceae within corals can lead to coral bleaching (breakdown of symbiosis), reduced translocation of autotrophically-derived nutrients to the host, reduced reproduction and mortality^29,30,65^, and similar responses are possible in symbiotic foraminifera^22^, jellyfish^64^ and ascidians^61^. The free-living Symbiodiniaceae in culture and in the environment alternate between motile and non-motile forms^66^. This mobility facilitates dispersal and infection of host recruits, which are initially symbiont free for most coral species^67^. Impacts of PSII herbicides on populations of free-living Symbiodiniaceae could limit the onset of mutualistic endosymbiosis in corals and other symbiotic invertebrates that have a critical reliance on their symbionts for autotrophic energy acquisition. Free-living Symbiodiniaceae also represent a reservoir of symbionts for bleached coral hosts that are needed for rapid recovery and survival following thermal bleaching events^31,68^. Indeed, free-living Symbiodiniaceae are widespread in the ocean, with a recent study detecting this family in over 90% of sampling sites and making up 0.1% of total eukaryotic reads in tropical and sub-tropical waters^31^. Impacts by herbicides on these highly diverse and abundant free-living populations of Symbiodiniaceae may therefore have ecological consequences beyond the risk to their role in invertebrate symbiosis.

### Implications for water quality guideline values

The Australian and New Zealand marine WQGVs for all herbicides tested here (apart from the reference herbicide diuron) are of low reliability due to lack of appropriate marine toxicity threshold data^39,41–43^. The SGR toxicity thresholds for cultured *C. goreaui* represent the first toxicity dataset for Symbiodiniaceae that is suitable for application to water quality guideline derivation. The comparatively slow growth rate of *C. goreaui* (common for all Symbiodiniaceae) meant that the chronic growth assay for this species needed to be longer than the standard 3- or 4-day exposure applied for most algal species. Consequently, the toxicity thresholds identified here could be considered relatively conservative and appropriate for comparison against long in situ exposure durations. NEC and EC_10_ toxicity thresholds are the preferred data for inclusion in Species Sensitivity Distributions (SSDs) used to derive WQGVs^45^ and these are compared in Table 5 against existing and proposed WQGVs for each of the herbicides. Australian and New Zealand WQGVs are currently derived to protect 99, 95, 90 and 80% (PC99, 95, 90 and 80, respectively) of species in marine and freshwater ecosystems^41^, and Table 5 compares SGR endpoints rather than those for ΔF/F_m_′, as inhibition of growth is currently considered to better represent an ecologically relevant impact^45^.

**Table 5:** Comparison of toxicity thresholds derived here vs Australian WQGVs.

Herbicide	This study		Proposed WQGV				Current WQGV			
	NEC	EC_10_	99%	95%	90%	80%	99%	95%	90%	80%
Diuron	2.75	2.54	0.43	0.67	0.86	1.2	0.2			
Bromacil	16.6	18.3	0.23	1.1	2.2	4.8	180			
Metribuzin	23.6	22.3	2.0	2.7	3.1	3.9	NA			
Propazine	45.1	50.8	2.2	4.6	6.4	9.2	NA			
Hexazinone	71.7	78.7	1.8	2.5	3.1	4.0	75			
Simazine	320	257	28	63	84	130	0.2	3.2	11	35
Tebuthiuron	107	138	4.7	11	17	26	0.02	2.2	20	160
Haloxyfop	> 2980	590	2000	3400	6100	NA				
Imazapic	> 165,000	0.049	0.44	1.2	3.6	NA				

Comparisons of toxicity thresholds of EC_10_ and NEC for specific growth rate (SGR) of *Cladocopium goreaui* in response to nine herbicides with current guideline values^41^ and proposed guideline values^39,42,43^ derived for 99, 95, 90 and 80% species protection (PC99, PC95, PC90, PC80 respectively). All concentrations are in µg L^−1^. NA denotes no current WQGV.

The SGR NECs and EC_10_s for all nine herbicides were greater than the proposed WQGVs (Table 5), indicating the proposed WQGVs would be protective of *C. goreaui* growth*.* The current WQGVs^41^ were proposed three decades ago and current guideline values for bromacil and hexazinone would not be protective of *C. goreaui.* However, the SGR toxicity thresholds for *C. goreaui*, in combination with other similar data for the tropical marine cryptophyte *R. salina*^23^, the diatom *C. muelleri*^38^ and the coral *Acropora tenuis*^69^ will contribute to improving the reliability of WQGVs and their relevance to tropical marine ecosystems such as the GBR. The SGR toxicity thresholds reported here for *C. goreaui* were all higher than concentrations detected in the GBR of up to 0.778 µg diuron L^−1^ over time averaged month-long passive sampler deployments^12^. Additionally, the ΔF/Fm′ toxicity thresholds for *C. goreaui*, except for diuron, were also higher than concentrations reported in the GBR^12^. However, improving WQGVs for alternative pesticides (such as the herbicides in this study) will improve confidence in the application of ms-PAF to predict the total toxicity of all pesticides detected in water quality monitoring programs. For example, over 80% of water samples taken in the GBRCA between 2011 and 2015 contained between 2 and 20 quantifiable pesticides for samples collected in the GBRCA^8^. Including the contribution of all pesticides to ecological risk using ms-PAF results in more reported exceedances of WQGVs^12^, and is the preferred method in monitoring programs that inform agricultural management practices adjacent to sensitive marine habitats such as the GBR^6^.

## Conclusion

The toxicity growth thresholds for alternative herbicides to the free-living form of the common coral symbiont *C. goreaui* were reported for the first time. The seven PSII herbicides all inhibited photosynthetic efficiency (ΔF/F_m_′), leading to reduced SGR over the 14-d exposure period. The sensitivity of ΔF/F_m_′ to PSII herbicides was on average 4.4-fold greater than for SGR, but the clear mechanistic link and consistent relationship between inhibition of ΔF/F_m_′ and SGR indicates that inhibition of ΔF/F_m_′ should be considered a biologically relevant toxicity endpoint for PSII herbicides to marine microalgae^23,37,38^. The non-PSII herbicides haloxyfop and imazapic did not affect SGR in *C. goreaui* at very high concentrations indicating these individual herbicides do not pose a significant risk to this species. The effects of PSII herbicides on free-living *C. goreaui* occurred at similar concentrations as shown for Symbiodiniaceae *in hospite*, indicating cultures of this species are relevant proxies for both free-living and symbiotic forms of this species. As mutualistic endosymbiotic partners with scleractinian corals, inclusion of toxicity data for Symbiodiniaceae to relevant contaminants such as alternative herbicides will improve our confidence that future WQGVs are adequate to inform risk assessments for tropical marine ecosystems. While this study targeted some of the most frequently detected alternative herbicides in GBR waters, there remains several pesticides, including insecticides and fungicides, with no current WQGVs and further testing is needed to address this.

## Materials and methods

### Test species and culture conditions

A monoclonal strain of *Cladocopium goreaui* (formerly *Symbiodinium* clade C1^70^) was isolated in 2010 from coral *Acropora tenuis* near Magnetic Island in Queensland, Australia. In house cultures (Australian Institute of Marine Science ID: SCF 055-01.10) were maintained in sterile 75-mL culture flasks in IMK growth media prepared with 0.2 µm-filtered seawater (pH = 7.8 ± 0.5; salinity = 32.5 ± 0.7 psu; Wako Chemicals USA, Richmond) and incubated in Steridium environmental chambers at 14:10 h light:dark cycles with an irradiance of 60–75 μmol quanta m^−2^ s^−1^ (Sylvania FHO24W/T5/865) at 27 ± 0.45 °C. Fortnightly 10% inoculations were performed on cultures to consistently use 14-days old cultures in toxicity bioassays.

### Herbicide stock preparation

Herbicide stock solutions were prepared with PESTANAL analytical grade products (HPLC ≥ 98%) and purchased from Sigma-Aldrich (NSW, Australia), including: diuron (CAS 330-54-1), bromacil (CAS 314-40-9), haloxyfop-p-methyl (CAS 72619-32-0), hexazinone (CAS 51235-04-2), imazapic (CAS 104098-48-8), metribuzin (CAS 21087-64-9), propazine (CAS 139-40-2), simazine (CAS 122-34-9), and tebuthiuron (CAS 34014-18-1). Stock solutions (5–600 mg L^−1^) were prepared in Milli-Q water or filtered seawater (FSW) in 500 mL or 1 L autoclaved Schott bottles. Diuron and metribuzin were dissolved using the carrier solvent ethanol (EtOH; ≤ 0.002% v/v in all exposure treatments). Haloxyfop and simazine were dissolved in the carrier solvent dimethyl sulfoxide (DMSO; ≤ 0.006% v/v in all exposure treatments). No solvent carrier was used in the preparation of the remaining herbicide stock solutions.

### Bioassay protocol

Cultures of 14-days old *C. goreaui* in exponential growth phase at a starting density of 1.7–2.7 × 10^4^ cells mL^−1^ were used in all bioassays. The required algae inoculum was transferred to 50 mL polypropylene conical centrifuge tubes (3–6 replicates per treatment) containing 30 mL of IMK media for 14-days exposure tests. Each 50 mL centrifuge tube was dosed with a range of herbicide concentrations. Treatments included control (no herbicide) and herbicide reference (diuron, 6 µg L^−1^). *C. goreaui* cells were incubated for 14 days at 130 rpm in a refrigerated incubator shaker (Thermoline Scientific) at 27 ± 1 °C on a 14:10 h light:dark cycle under 60–75 μmol quanta m^−2^ s^−1^. Samples were randomized every 1–2 days to ensure similar light conditions for all replicate samples.

### Cell density counts and specific growth rate

Aliquots (1 mL) of treatment samples were fixed in glutaraldehyde (0.5% v/v final concentration) and surfactant (Pluronic F68; 0.1% v/v final concentration) to reduce clumping of cells as per Marie et al.^44^. A flow cytometer (BD Accuri C6, BD Biosciences, CA, USA) equipped with one red and blue laser (488 nm, 50 mW maximum solid state; 640 nm, 30 mW diode) was used to quantify cell density as per Trenfield et al.^71^ at several timepoints during each bioassay. A lag phase of 3–4 days was typically observed for *C. goreaui*; therefore, day 4 was considered the first timepoint for measuring cell density over the logarithmic growth phase. Subsequent timepoints (typically, day 4, 7, 10 and 14) were chosen due to availability of instrument. Flow rate was set to 35 µL min^−1^, 16-µm core size and sample volume of 50 µL. A fixed gating was used to exclusively measure *C. goreaui*, minimizing quantification of microbes and degraded chloroplasts of senescing cells. Viable *C. goreaui* cells represented 70–90% of all particles identified in samples over 14 days. Specific growth rates (SGR) were expressed as the logarithmic increase in cell density from day i (t_i_) to day j (t_j_) as per Eq. (1), where SGR_i-j_ is the specific growth rate from time i to j, X_j_ is the cell density at day j and X_i_ is the cell density at day i^58^. SGR relative to the control treatment was used to derive modelled chronic effect concentration values for growth inhibition. Test acceptability was confirmed if the SGR of control replicates was ≥ 0.1 day^−1^ (1 doubling/week) and had a coefficient of variation (CV) ≤ 10% for control SGR^58,72–75^.

1 $${\text{SGR}}_{{\text{i - j}}} = \frac{{\ln {\text{X}}_{{{\text{j}} }} - \ln {\text{X}}_{{\text{i}}} }}{{{\text{t}}_{{\text{j}}} - {\text{t}}_{{\text{i}}} }} ({\text{day}}^{ - 1} )$$

### Chlorophyll fluorescence

Chlorophyll fluorescence was used as a proxy to measure photosynthetic health of *C. goreaui* using pulse amplitude modulation fluorometry (microscopy imaging PAM, Walz, Germany) to determine effective quantum yield (ΔF/F_m_′) after 14 days herbicide exposure tests. ΔF/F_m_′ was calculated from light adapted minimum fluorescence (F) and maximum fluorescence (F_m_′) measurements, following Eq. (2) from Schreiber et al.^76^. ΔF/F_m_′ was measured from three replicates per treatment, including the control and reference toxicant. A minimum of three cells was measured with acceptable control treatment values. The observed control measurements for ΔF/F_m_′ were within acceptable limits (ΔF/F_m_′ > 0.30) as per Hennige et al.^72^ and Karim et al.^77^.

2 $$\Delta F/Fm^{\prime} = \frac{{Fm^{\prime} {-} F}}{{Fm^{\prime}}}$$

Percent inhibition was calculated relative to controls as per Eq. (3). Inhibition of ΔF/F_m_′ suggests reduced energy conversion in PSII of the organism under a known light intensity. These measurements were used to compare photoinhibition between the controls and respective treatments. Three to six replicates at 6 μg L^−1^ diuron were included in each bioassay to estimate the consistency of inhibitory responses of cells. Microscopy iPAM settings were: actinic light = 1, measuring light = 10–12, gain = 3, damp = 2, actinic light width = 180 s and saturation pulse intensity = 2.

3 $$\% {\text{ Inhibition}} = \frac{{{\text{X}}_{{{\text{control}}}} - {\text{X}}_{{{\text{treatment}}}} }}{{{\text{X}}_{{{\text{control}}}} }} \times 100$$

### Physico-chemical and herbicide analyses

Physical and chemical characteristics of each treatment were measured at 0 days and 14 days including pH and salinity (LAQUAact-PC110 Meter, HORIBA Scientific) and dissolved oxygen (HQ30D Portable Meter, HACH) as per Thomas et al.^23^. Temperature was logged in 10-min intervals over the duration of the test (HOBO, Onset). Herbicide analytical samples were taken at 0 day and 14 days. Aliquots (1 mL) for chemical analysis were transferred into 1.5 liquid chromatography amber glass vials and spiked with surrogate standards (i.e. diuron-d6, hexazinone-d6, metribuzin-d3, simazine-D10, propazine-D6, bromacil-D3, haloxyfop-D4, and imazapic D7) with a 10 ng mL^−1^ final concentration of the surrogate standard. The herbicide and degradation product concentrations were determined by HPLC–MS/MS using an SCIEX Triple Quad 6500 QTRAP mass spectrometer (SCIEX, Concord, Ontario, Canada) equipped with an IonDrive Turbo V ion source using a TurboIonSpray probe^23,78,79^. Data acquisition and quantification were performed on MultiQuant 3.0 software by Sciex. Compound identification and confirmation were achieved using retention times and via comparison of SRM transition intensity ratios between the sample and isotopically labelled standard in the same run. The geometric mean from start and end concentrations (time-weighted average) was assigned as the ‘measured’ concentration in that sample. The average loss from these measured concentrations was then applied to all nominal concentrations as per Thomas et al.^23^.

### Data analyses

Measured concentrations were used for all concentration–response modelling and toxicity threshold estimates (Table S1). Specific growth rate and photosynthetic inhibition were calculated as percent inhibition relative to the solvent control or IMK media control (in bioassays when no solvent carrier was used). Herbicide concentrations that inhibited 10 and 50% of growth or ΔF/F_m_′ relative to controls (EC_10_ and EC_50,_ respectively) were interpolated from modelled mean values using GraphPad Prism V 7.0 (GraphPad Software Inc., San Diego, USA). Relative potencies (ReP) were calculated for each herbicide (ReP = EC_50_ diuron/EC_50_ herbicide) against the reference toxicant diuron. The estimation of no effect concentrations (NEC) for SGR was calculated in R (V 3.6.1) as per Thomas et al.^23^. Proportional decline in SGR (1-inhibition) was modelled as a function of log concentration of each herbicide using a Bayesian non-linear gaussian model with the R package jagsNEC^80^. This model has been specifically developed to derive no effect concentrations (NECs) but also allows the estimation of EC_10_ and EC_50_ values and is adapted from Fox^81^. See Thomas et al.^23^ for further details. The linear regression of EC50s SGR vs ΔF/Fm′ was performed in SigmaPlot V14 (Systat Software, San Jose, CA).

## Supplementary Information

Supplementary Information.
