# Supplementary material for: Toxicity thresholds of nine herbicides to coral symbionts (Symbiodiniaceae)
Source: Sci Rep. 2021 Nov 4;11:21636. doi: 10.1038/s41598-021-00921-3 (PMC8568975; doi:10.1038/s41598-021-00921-3)
Supplement: Supplementary file 1 — Supplementary Information. [file 41598_2021_921_MOESM1_ESM.docx]

**Toxicity thresholds of nine herbicides to coral symbionts (Symbiodiniaceae)**

**Magena Marzonie^1,2^, *Florita Flores^1,2^, Nora Sadoun^1^, Marie C. Thomas^1^, Anais Valada-Mennuni^1^, Sarit Kaserzon^3^, Jochen F. Mueller^3^, Andrew P. Negri^1,2^**

^1^Australian Institute of Marine Science, Townsville, QLD 4810, Australia

^2^AIMS@JCU: Australian Institute of Marine Science and College of Marine and Environmental Sciences, James Cook University, Townsville, QLD 4810, Australia

^3^Queensland Alliance for Environmental Health Sciences (QAEHS), The University of Queensland, Woolloongabba, QLD 4102, Australia

*Email: f.flores@aims.gov.au

**Table S-1. Summary of the chemical properties, stock solutions, and nominal and measured concentrations of herbicides used in this study**. A solvent carrier was used in the diuron (ethanol), metribuzin (ethanol), simazine (DSMO), and haloxyfop (DSMO) toxicity tests, and solvent carrier controls (SC) were used for those tests. The solvents were chosen based on their relative ability to dissolve the herbicides at the concentrations required. Seawater controls (SWC) were used for all tests. Log K_OW_ values from PubChem (https://pubchem.ncbi.nlm.nih.gov/). Solubilities in water (at 20 °C) are compiled using the Pesticide Properties Database (https://sitem.herts.ac.uk/aeru/ppdb/). Measured concentrations are the average of initial and final concentration in each test. FSW denotes filtered seawater.

| **Herbicide** | **Solubility (mg L^-1^)** | **log KOW** | **Stock solution (mg L^-1^)** | **FSW/Milli-Q** | **Nominal concentration (µg L^-1^)** | | **Measured concentration (µg L^-1^)** | |
| --- | --- | --- | --- | --- | --- | --- | --- | --- |
|  |  |  |  |  | Growth test | PAM test | Growth test | PAM test |
| *Inhibition of photosynthesis at PSII* | | | | | | | | |
| Diuron | 35.6 | 2.68 | 10 | Milli-Q | SWC; SC; 0.5; 1; 2; 4; 6; 10 | SWC; SC; 0.5; 1; 2; 4; 6; 10 | 0; 0; 0.49; 0.98; 1.97; 3.94; 5.91; 9.85 | 0; 0; 0.49; 0.98; 1.97; 3.94; 5.91; 9.85 |
| Bromacil | 815 | 2.11 | 10 | Milli-Q | SWC; 5; 10; 15; 20; 30; 40; 50 | SWC; 5; 10; 15; 20; 30; 40; 50 | 0; 4.59; 9.18; 13.76; 18.35; 27.53; 36.70; 45.88 | 0; 4.59; 9.18; 13.76; 18.35; 27.53; 36.70; 45.88 |
| Hexazinone | 33000 | 1.85 | 5 | Milli-Q | SWC; 3; 6; 12; 25; 75; 150 | SWC; 3; 6; 12; 25; 75; 150 | 0; 3.81; 7.61; 15.22; 31.71; 95.14; 190.29 | 0; 3.81; 7.61; 15.22; 31.71; 95.14; 190.29 |
| Metribuzin | 1165 | 1.7 | 10 | Milli-Q | SWC; SC; 0.3; 1; 3; 10; 30; 100 | SWC; SC; 0.3; 1; 3; 10; 30; 100 | 0; 0; 0.28; 0.94; 2.81; 9.35; 28.05; 93.52 | 0; 0; 0.28; 0.94; 2.81; 9.35; 28.05; 93.52 |
| Propazine | 8.6 | 2.93 | 5 | FSW | SWC; 20; 40; 80; 120; 160; 180; 200 | SWC; 20; 40; 80; 120; 160; 180; 200 | 0; 10.23; 20.46; 40.92; 61.38; 81.83; 92.06; 102.29 | 0; 10.23; 20.46; 40.92; 61.38; 81.83; 92.06; 102.29 |
| Simazine | 5 | 2.18 | 5 | FSW | SWC; SC; 10; 30; 100; 150; 200; 300; 600 | SWC; SC; 10; 30; 100; 150; 200; 300; 600 | 0; 0; 15.22; 45.67; 152.25; 228.37; 304.50; 456.75; 913.50 | 0; 0; 15.22; 45.67; 152.25; 228.37; 304.50; 456.75; 913.50 |
| Tebuthiuron | 2500 | 1.79 | 10 | Milli-Q | SWC; 18.75; 37.5; 75; 150; 300; 600 | SWC; 18.75; 37.5; 75; 150; 300; 600 | 0; 10.72; 21.45; 42.90; 85.79; 171.58; 343.16 | 0; 10.72; 21.45; 42.90; 85.79; 171.58; 343.16 |
| *Inhibition of acetyl-CoA carboxylase (ACCase)* | | | | | | | | |
| Haloxyfop | 1.6 | 4.2 | 100 | FSW | SWC; SC; 1,000; 2,000; 4,000; 6,000; 8,000; 12,000 | SWC; SC; 1,000; 2,000; 4,000; 6,000; 8,000; 12,000 | 0; 0; 248; 496; 992; 1,488; 1,985; 2,977 | 0; 0; 248; 496; 992; 1,488; 1,985; 2,977 |
| *Inhibition of acetohydroxyacid synthase (AHAS)* | | | | | | | | |
| Imazapic | 2230 | 0.393 | 600 | FSW | SWC; 5,000; 10,000; 20,000; 40,000; 60,000; 120,000; 240,000; 480,000; 600,000 | SWC; 5,000; 10,000; 20,000; 40,000; 60,000; 120,000; 240,000; 480,000; 600,000 | 0; 1,374; 2,747; 5,495; 10,989; 16,484; 32,968; 65,936; 131,872; 164,840 | 0; 1,374; 2,747; 5,495; 10,989; 16,484; 32,968; 65,936; 131,872; 164,840 |
|  | | | | | | | | |

**Table S-2. Summary of water quality measurements.** Physico-chemical measurements of each treatment and test measured at test initiation (0 h) and test finalization (14 d) including pH, salinity, and dissolved oxygen (DO). Temperature was logged in 10-min intervals over the total test duration. NA denotes data not measured.

| **Herbicide** | **Nominal concentration (µg L^-1^)** | **Test initiation (0 h)** | | | | **Test finalization (14 d)** | | | |  |
| --- | --- | --- | --- | --- | --- | --- | --- | --- | --- | --- |
|  |  | **pH** | **Salinity** | **DO** | | **pH** | **Salinity** | **DO** | | **Temperature (mean ± SD)** |
|  |  | units | psu | mg L^-1^ | % | units | psu | mg L^-1^ | % | °C |
| Diuron | 0 | 7.59 | 30.7 | 7.93 | 94.3 | 7.5 | 31 | 8.28 | 99.4 | 27.12 ± 0.37 |
|  | 0.5 | 7.58 | 31 | 7.93 | 94.3 | 7.49 | 31 | 8.15 | 98.9 |  |
|  | 1 | 7.58 | 31 | 7.84 | 93.4 | 7.48 | 31 | 8.1 | 98.7 |  |
|  | 2 | 7.59 | 31.2 | 7.91 | 94.1 | 7.48 | 31 | 8.1 | 98.8 |  |
|  | 4 | 7.58 | 31 | 7.85 | 93.5 | 7.47 | 31 | 7.95 | 96.9 |  |
|  | 6 | 7.58 | 30.9 | 7.86 | 93.5 | 7.47 | 31 | 7.9 | 96 |  |
|  | 10 | 7.58 | 31 | 7.81 | 92.6 | 7.45 | 31 | 7.89 | 95.3 |  |
| Bromacil | 0 | 7.59 | 33.1 | 8.09 | 93.7 | 8.39 | 32.8 | 7.09 | 79.7 | 26.29 ± 0.71 |
|  | 5 | 7.46 | 32.9 | 7.46 | 92.8 | 8.39 | 32.7 | 7.14 | 80.7 |  |
|  | 10 | 7.52 | 32.9 | 7.52 | 92.3 | 8.39 | 32.7 | 7.1 | 80.1 |  |
|  | 15 | 7.53 | 32.9 | 7.53 | 91.6 | 8.22 | 32.8 | 7.15 | 81.6 |  |
|  | 20 | 7.57 | 32.8 | 7.57 | 91.8 | 7.92 | 32.8 | 7.51 | 85.6 |  |
|  | 30 | 7.59 | 32.8 | 7.59 | 91.5 | 7.57 | 32.7 | 7.74 | 88.3 |  |
|  | 40 | 7.58 | 32.8 | 7.58 | 91.4 | 7.56 | 32.7 | 7.96 | 90.7 |  |
|  | 50 | 7.6 | 32.9 | 7.6 | 91.8 | 7.53 | 32.9 | 8.02 | 91.5 |  |
|  | ^a^Diuron | 7.57 | 32.8 | 7.57 | 92.6 | 7.58 | 32.9 | 7.8 | 89 |  |
| Hexazinone | 0 | 7.81 | 31.8 | 8.05 | 96.4 | 8.53 | 32.2 | 8.02 | 96.1 | 26.90 ± 0.36 |
|  | 3 | 7.72 | 31.8 | 8.03 | 96.2 | 8.64 | 32.1 | 8.03 | 96.6 |  |
|  | 6 | 7.73 | 31.8 | 8.11 | 97.2 | 8.65 | 32.2 | 7.9 | 95.4 |  |
|  | 12 | 7.78 | 31.8 | 8.07 | 97.1 | 8.66 | 32.2 | 7.91 | 95.2 |  |
|  | 25 | 7.79 | 31.8 | 8.02 | 96.4 | 8.53 | 32.3 | 7.9 | 95.5 |  |
|  | 75 | 7.84 | 31.8 | 8.03 | 96.5 | 8.11 | 32.3 | 7.82 | 94.4 |  |
|  | 150 | 7.93 | 31.8 | 8.13 | 97.6 | 7.73 | 32.3 | 7.88 | 95 |  |
|  | Diuron | 7.93 | 31.7 | 8.01 | 96.3 | 7.66 | 32.3 | 7.77 | 93.4 |  |
| Metribuzin | 0 | 8.14 | 32 | 7.71 | 95.2 | 8.81 | 32.5 | 8.1 | 96.2 | 27.11 ± 0.39 |
|  | 0.3 | 8.11 | 32 | 7.73 | 94.7 | 8.78 | 32.6 | 8.06 | 95.1 |  |
|  | 1 | 8.08 | 32 | 7.73 | 94.3 | 8.82 | 32.3 | 8.07 | 95.5 |  |
|  | 3 | 8.11 | 32.1 | 7.74 | 94.7 | 8.8 | 32.3 | 8.05 | 95.3 |  |
|  | 10 | 8.08 | 32.1 | 7.74 | 94.4 | 8.73 | 32.5 | 8.02 | 94.9 |  |
|  | 30 | 8.06 | 32 | 7.74 | 94.3 | 8.04 | 32.4 | 7.98 | 94.2 |  |
|  | 100 | 8.11 | 32.1 | 7.75 | 94.9 | 7.69 | 32.5 | 7.93 | 93.2 |  |
|  | Diuron | 8.07 | 32 | 7.72 | 94.4 | 7.69 | 32.6 | 7.99 | 94.8 |  |
| Propazine | 0 | 7.68 | 33 | 7.78 | 90.1 | 8.26 | 32.8 | 6.98 | 78.2 | NA |
|  | 20 | 7.65 | 32.8 | 7.85 | 90.8 | 8.33 | 32.7 | 7 | 79.1 |  |
|  | 40 | 7.65 | 32.8 | 7.86 | 90.9 | 8.32 | 32.6 | 7.02 | 79.5 |  |
|  | 80 | 7.64 | 32.8 | 7.8 | 90.5 | 8.11 | 32.8 | 7.06 | 80.6 |  |
|  | 120 | 7.64 | 32.7 | 7.76 | 89.8 | 7.73 | 32.8 | 7.41 | 84.5 |  |
|  | 160 | 7.64 | 32.8 | 7.87 | 91 | 7.7 | 32.7 | 7.62 | 86.8 |  |
|  | 180 | 7.62 | 32.8 | 7.8 | 90 | 7.66 | 32.7 | 7.76 | 88.4 |  |
|  | 200 | 7.63 | 32.8 | 7.86 | 91 | 7.66 | 32.8 | 7.82 | 89.1 |  |
|  | Diuron | 7.63 | 32.8 | 7.82 | 90.5 | 7.61 | 32.9 | 7.75 | 88.5 |  |
| Simazine | 0 | 7.44 | 32.7 | 8.05 | 94.7 | 9.67 | 32.4 | 8.31 | 98.9 | NA |
|  | 10 | 7.46 | 33.2 | 7.88 | 93.5 | 9.75 | 33.4 | 8.08 | 96.7 |  |
|  | 30 | 7.63 | 33.3 | 7.92 | 94.2 | 9.03 | 33.5 | 8.07 | 96.7 |  |
|  | 100 | 7.57 | 33.2 | 7.86 | 93.2 | 8.38 | 33.4 | 7.82 | 93.5 |  |
|  | 150 | 7.51 | 33.2 | 7.8 | 92.7 | 8.02 | 33.4 | 7.82 | 93.7 |  |
|  | 200 | 7.58 | 33.2 | 7.74 | 92.2 | 7.71 | 33.4 | 7.79 | 93.3 |  |
|  | 300 | 7.4 | 33.2 | 7.72 | 92 | 7.59 | 33.4 | 7.76 | 92.9 |  |
|  | 600 | 7.38 | 33.2 | 7.63 | 90.7 | 7.55 | 33.5 | 7.77 | 92.8 |  |
|  | Diuron | 7.38 | 33 | 7.71 | 91.3 | 7.54 | 33.3 | 8.31 | 98.9 |  |
| Tebuthiuron | 0 | 7.57 | 32.3 | 7.22 | 89.1 | 7.77 | 33.2 | 8.08 | 106 | 26.29 ± 0.71 |
|  | 18.75 | 7.54 | 32.5 | 7 | 86.4 | 7.53 | 33.4 | 7.88 | 98.3 |  |
|  | 37.5 | 7.34 | 31.7 | 7.23 | 89.1 | 7.49 | 33.6 | 7.79 | 97.3 |  |
|  | 75 | 7.57 | 32.3 | 7.47 | 92.1 | 7.7 | 33.6 | 7.9 | 98.8 |  |
|  | 150 | 7.52 | 32.3 | 7.43 | 91.4 | 7.53 | 33.6 | 7.78 | 97.4 |  |
|  | 300 | 7.57 | 32.4 | 7.31 | 90 | 7.48 | 33.6 | 7.73 | 96.7 |  |
|  | 600 | 7.58 | 32.4 | 7.48 | 92.2 | 7.45 | 33.6 | 7.72 | 96.6 |  |
|  | Diuron | 7.58 | 32.4 | 7.49 | 92.3 | 7.72 | 31.5 | 7.98 | 92.7 |  |
| Haloxyfop | 0 | 8.1 | 31.2 | 7.87 | 94.3 | 8.57 | 32.2 | 7.52 | 88 | 27.40 ± 0.36 |
|  | 1,000 | 8 | 31.7 | 7.76 | 93.3 | 8.61 | 32.3 | 7.33 | 85.8 |  |
|  | 2,000 | 7.98 | 31.8 | 7.81 | 94 | 8.65 | 32.3 | 7.1 | 83.2 |  |
|  | 4,000 | 7.98 | 31.8 | 7.84 | 94.2 | 8.67 | 32.3 | 7.55 | 88.6 |  |
|  | 6,000 | 7.95 | 31.9 | 7.82 | 94 | 8.64 | 32.3 | 7.24 | 85.1 |  |
|  | 8,000 | 7.96 | 32 | 7.78 | 93.4 | 8.63 | 32.4 | 7 | 82.5 |  |
|  | 12,000 | 7.96 | 32.1 | 7.7 | 92.4 | 8.62 | 32.5 | 7.26 | 86 |  |
|  | Diuron | 7.9 | 31 | 7.76 | 92.4 | 7.92 | 32.2 | 7.55 | 89.6 |  |
| Imazapic | 0 | 7.8 | 33 | 8.13 | 98.2 | 7.64 | 33.4 | 7.89 | 98.6 | 27.43 ± 0.36 |
|  | 5,000 | 7.81 | 33 | 8.05 | 98 | 7.65 | 33.5 | 7.68 | 96 |  |
|  | 10,000 | 7.8 | 33 | 8.02 | 97.3 | 7.61 | 33.5 | 7.69 | 96.2 |  |
|  | 20,000 | 7.76 | 33 | 7.97 | 97 | 7.56 | 33.5 | 7.7 | 96.4 |  |
|  | 40,000 | 7.68 | 33 | 7.92 | 96.6 | 7.5 | 33.5 | 7.69 | 96.2 |  |
|  | 60,000 | 7.52 | 33 | 7.91 | 97 | 7.48 | 33.5 | 7.73 | 96.7 |  |
|  | 120,000 | 7.19 | 33 | 7.84 | 96.3 | 7.36 | 33.5 | 7.69 | 96.2 |  |
|  | 240,000 | 6.76 | 33.1 | 7.79 | 95.7 | 7.07 | 33.6 | 7.62 | 95.4 |  |
|  | 480,000 | 5.96 | 33 | 7.73 | 94.6 | 6.7 | 33.5 | 7.7 | 96.3 |  |
|  | 600,000 | 5.26 | 33 | 7.68 | 94.4 | 5.73 | 33.5 | 7.79 | 97.5 |  |
|  | Diuron | 7.78 | 33 | 7.79 | 95.4 | 7.31 | 33.5 | 7.75 | 97.1 |  |

^a^Reference treatment (diuron; 6 µg L^-1^)
